# Ameliorative effects of *Brassica oleracea* var. *viridis* ethanol extract and fractions on cimetidine-induced reproductive toxicity in male Wistar rats

**DOI:** 10.3389/fvets.2025.1645967

**Published:** 2025-08-25

**Authors:** Emmanuel Orire Ikuomola, Daniel Udofia Owu, Victor Otu Oka, Olufunke Onaadepo, Felix Nnaemeka Ugwu, Patrick Maduabuchi Aja

**Affiliations:** ^1^Department of Physiology, Faculty of Biomedical Sciences, Kampala International University Western Campus, Bushenyi, Uganda; ^2^Department of Physiology, Faculty of Biomedical Sciences, Kampala International University, Kampala, Tanzania; ^3^Department of Biochemistry, Faculty of Biomedical Sciences, Kampala International University Western Campus, Bushenyi, Uganda

**Keywords:** *Brassica oleracea*, cimetidine, collard greens, oxidative stress, hormonal regulation

## Abstract

**Background:**

Male infertility is a global health issue, with pharmaceutical agents such as cimetidine contributing significantly to gonadotoxicity through antiandrogenic and oxidative mechanisms. The search for natural protective agents has highlighted *Brassica oleracea* var. *viridis* (collard greens) for its antioxidant and endocrine-modulating properties.

**Objectives:**

This study evaluated the protective effects of *Brassica oleracea* var. *viridis* (collard greens) ethanol extract and its solvent fractions on cimetidine-induced reproductive toxicity in male Wistar rats, focusing on body/organ weights, hormonal profiles, antioxidant enzyme activities, and testicular histoarchitecture.

**Methods:**

Thirty-five rats were divided into seven groups: control, cimetidine (120 mg/kg), ethanol extract (200 mg/kg), and cimetidine + fractions (aqueous, butanol, and hexane). After 8 weeks oral administration of extracts/fractions, sperm parameters, serum hormones (LH, FSH, and testosterone), oxidative stress markers (catalase, SOD, and MDA), and histopathology were assessed.

**Results:**

Cimetidine significantly reduce follicle stimulating hormone (FSH), luteinizing hormone (LH) and testosterone levels (*p* < 0.05) while increasing oxidative stress, as evidenced by elevate malondialdehyde (MDA) superoxide dismutase (SOD) and catalase (CAT). It also caused distortion of the testicular architecture. Treatment with the ethanol extract (ELEBO) and solvent fractions restored hormonal balance and antioxidant activity. Histological analysis revealed preserved testicular architecture in treated groups compared to the degeneration observed in the cimetidine-induced group.

**Conclusion:**

*Brassica oleracea* var. *viridis* exhibits significant protective effects against cimetidine-induced reproductive toxicity through hormonal regulation, antioxidative mechanisms, and tissue preservation. The ethanol extract of *Brassica oleracea* (ELEBO) showed the most potent activity, supporting its potential use as a therapeutic adjunct in male infertility linked to pharmaceutical exposures.

## 1 Introduction

**Figure 1:**
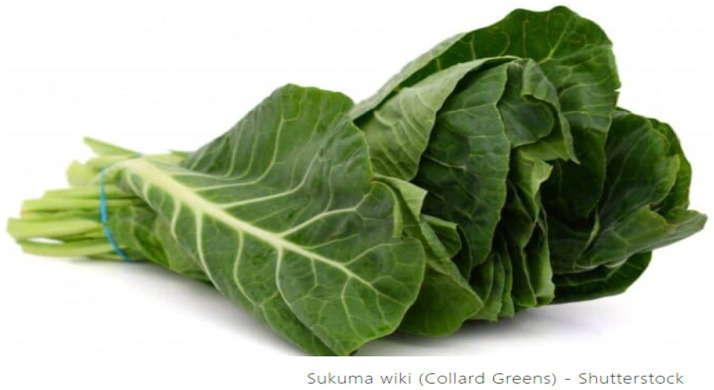
Collard green (*Brassica* *oleracea*) (37).

Male infertility is a growing global public health concern, accounting for nearly 50% of all infertility cases and affecting over 17% of the reproductive age population worldwide, according to the World Health Organization (1). In sub-Saharan Africa and Uganda in particular the burden of infertility remains substantial, with recent studies reporting prevalence rates as high as 15% among couples, with an overall infertility rate of 6.4% in Uganda between 2006 and 2016, with male-related factors such as sexually transmitted infections (STIs), lifestyle choices, and drug-induced gonadotoxicity being significant contributors (2, 3). Among the various contributors to male infertility, drug-induced testicular toxicity has emerged as a significant yet often overlooked factor. Cimetidine, an H₂-receptor antagonist commonly prescribed for peptic ulcers, has been widely implicated in male reproductive dysfunction. Although effective for gastrointestinal conditions, its prolonged use especially without medical supervision due to its affordability and over the counter availability poses serious reproductive risks. Cimetidine exerts antiandrogenic and antihistaminic effects that impair testosterone synthesis, disrupt spermatogenesis, and cause histopathological damage to testicular tissue (4–6). Cimetidine’s mechanism of reproductive toxicity includes competitive inhibition of dihydrotestosterone (DHT) binding at androgen receptors in the hypothalamus and pituitary, leading to hormonal imbalance and adverse outcomes such as reduced libido, impotence, oligozoospermia, and testicular degeneration (7–10). Although these deleterious effects on male reproductive health are documented, cimetidine remains widely used, including among males of reproductive age, underscoring the need for safe and effective strategies to mitigate its toxicity. In this context, there is growing scientific interest in exploring plant based therapies with antioxidative and hormone-modulating potential. One such candidate is *Brassica oleracea* var. *viridis* (commonly known as collard greens) as shown in Figure 1, a cruciferous vegetable that is widely cultivated and consumed in the western regions of Uganda. This vegetable holds a prominent place in local ethnomedicine, traditionally used to address male fertility challenges (11, 12). Its pharmacological potential stems from its rich profile of bioactive compounds, including polyphenols, flavonoids, isothiocyanates (such as sulforaphane), and indole-3-carbinol compounds known for their antioxidative, anti-inflammatory, and endocrine-regulating properties (12). These phytochemicals are believed to neutralize reactive oxygen species (ROS), reduce oxidative stress in reproductive tissues, and restore hormonal balance, making *B. oleracea* var. *viridis* a scientifically plausible candidate for counteracting drug-induced reproductive damage.

Despite its ethnobotanical relevance, the potential protective effects of *B. oleracea* against drug-induced reproductive toxicity, particularly cimetidine-related damage, remain underexplored. Given the antioxidant capacity of its bioactive constituents and its wide consumption in Uganda it is hypothesized that *B. oleracea* may ameliorate testicular dysfunction and hormonal disruption caused by cimetidine exposure (4, 13, 14).

This study therefore aims to investigate the therapeutic efficacy of ethanol extracts and solvent fractions of *Brassica oleracea* var. *viridis* in ameliorating cimetidine-induced reproductive toxicity in male Wistar rats. Key endpoints include changes in somatic and reproductive organ weights, serum hormonal profiles, testicular antioxidant enzyme activity, and histopathological alterations. Given its accessibility, affordability, and ethnobotanical relevance in Uganda, *B. oleracea* var. *viridis* could offer a valuable and sustainable intervention for reproductive health in resource-limited settings.

## 2 Methodology

### 2.1 Animal use and care

Thirty-five adult male Wistar rats (8–10 weeks old, weighing 180–220 g) were utilized in this study. The animals were obtained from the animal house of Kampala International University, Western Campus, Ishaka, Uganda. The rats were maintained in standard laboratory cages under controlled environmental conditions, with ad libitum access to water and a standard pellet diet. They were acclimatized to the laboratory environment for 1 week before the start of the experiment.

### 2.2 Reagents and materials

Cimetidine tablets (400 mg) used in this study was purchased from Cosmos Ltd., Nairobi, Kenya. For the extraction and fractionation process, a total of 5.5 L of 99% ethanol and 400 mL each of n-hexane and n-butanol were used. These solvents were purchased from God’s Grace Biomed Supply Ltd., Kampala, Uganda. The chemical-grade solvents were sourced from established manufacturers and used without further purification. Specifically, the n-hexane was obtained from Sigma-Aldrich (Cat. No. 296090), n-butanol from Merck (Cat. No. 101990), and 99% ethanol from Sigma-Aldrich (Cat. No. 24103). All reagents used in the study were of analytical grade and handled according to standard laboratory procedures.

### 2.3 Ethical clearance

An ethical clearance from the Research Ethics Committee (REC), Kampala International University Western Campus, Ishaka, Uganda was obtained. Also, REC number (KIU-2024-389) Uganda National for Science and Technology with registration number (HSF5192ES) was also obtained.

### 2.4 Plant collection and identification

Fresh leaves of *Brassica oleracea* var. *viridis* (collard greens) were collected from Bwejuragye, Ishaka Town, in the Bushenyi Local Government Area of Western Uganda. The plant material was transported to the Herbarium Unit of Mbarara University of Science and Technology for proper identification and verification. The Taxonomic and Morphological Identification Details of *Brassica oleracea* var. *viridis* as showed in Table 1. The species was identified and authenticated by Dr. Olet Eunice from the Department of Botany, Faculty of Biological Sciences, Mbarara University of Science and Technology, Uganda. A voucher specimen was deposited in the herbarium under the voucher number IOE-24-001.

**Table 1:** Showing taxonomic and morphological identification details of *Brassica oleracea* var. *viridis*.

Items	
Date of collection	03-09-2024
Collection number	IOE-24-001
Local name	Sukuma Wiki
Family name	Cruciferous family/Acephala group
Generic name	Chepkilumnda
Scientific name	*Brassica oleracea* var. viridis
Growth form	Rosette growth
Color	Dark green
Type of inflorescence	Raceme
Leaf shape	Ovate shape
Type of margin	Smooth or slightly undulating margin
Leaf venation	Palmate venation pattern
Pubescence	Glabrous (smooth and hairless) leaves
Leaf arrangement	Alternate leaf arrangement
Common plants around	Tomatoes, onions, garlic, beans, carrots
Type of ecosystem	Subtropical and tropical gardens
Locality	Bwejuragye, Ishaka town
Habitat	Subtropical and tropical areas

### 2.5 Preparation and extraction of *Brassica oleracea* (collard greens) leaves

Fresh leaves of *Brassica oleracea* (commonly known as collard greens) were thoroughly washed with distilled water to remove soil, debris, and other surface contaminants. The cleaned leaves were subsequently air-dried, ground into fine powder using a table grinder, and stored in airtight containers under dry conditions to prevent moisture absorption. Extraction was performed using a modified method based on the protocol (15). A total of 800 g of finely powdered leaf material was macerated in 5.5 L of 99% ethanol for 72 h, with appropriate labeling and protection from light and contamination. After maceration, the mixture was filtered, and the ethanol was removed from the filtrate using a rotary evaporator under reduced pressure. The resulting concentrate was further dried in an oven maintained at a temperature not exceeding 40 °C. The residual aqueous content was removed through lyophilization (freeze-drying) under vacuum. The final solvent-free extract was stored at 4 °C in a refrigerator until further use. The ethanol extraction yielded approximately 134 g of crude extract, corresponding to an extractive yield of 17%.

### 2.6 Fractionation procedure

For solvent partitioning, 100 g of the ethanol extract was reconstituted in 400 mL of distilled water and transferred into a separating funnel. An equal volume (400 mL) of n-hexane was added, and the mixture was vigorously shaken to allow for phase separation. The upper n-hexane layer was collected and labeled as the “n-hexane fraction.” The remaining aqueous phase was subsequently extracted with 400 mL of n-butanol using the same procedure. The resulting n-butanol layer was collected and labeled as the “n-butanol fraction.” The residual aqueous layer was lyophilized to obtain the “aqueous fraction.” The n-hexane and n-butanol fractions were concentrated by evaporation in an oven at 40 °C (16). Final yields were approximately 15 g (n-hexane fraction), 9 g (n-butanol fraction), and 120 g (aqueous fraction). All fractions were transferred to amber glass bottles and stored at 4 °C until needed for subsequent experimental procedures.

### 2.7 Stock solution of cimetidine

A stock solution was prepared by dissolving 5.2 g of cimetidine in 100 mL of distilled water. From this stock, a dose of 120 mg/kg of cimetidine was administered orally to each rat using an oral cannula. The dosing regimen for cimetidine administration was adapted from Liu et al. (17).

### 2.8 Experimental animal groupings

Group 1 received 2 mL/kg of distilled water for 8 weeks, group 2 received 200 mg/kg of ethanol extract for 8 weeks, group 3 received 120 mg/kg of cimetidine for 8 weeks, group 4 received 120 mg/kg of cimetidine + 200 mg/kg of aqueous extract for 8 weeks, group 5 received 120 mg/kg of cimetidine + 200 mg/kg of ethanol extract for 8 weeks, group 6 received 120 mg/kg of cimetidine + 200 mg/kg of n-butanol fraction for 8 weeks, and group 7 received 120 mg/kg of cimetidine + 200 mg/kg of n-hexane fraction for 8 weeks. The detailed study design is shown in Table 2.

**Table 2:** Experimental design.

Groups	Number of rats	Treatments	Number of days
Group 1 (control)	5	2 mL/kg of distilled water	8 weeks treated
Group 2 (ELEBO)	5	200 mg/kg of ethanol extract	8 weeks treated
Group 3 (CTD)	5	120 mg/kg cimetidine	8 weeks treated
Group 4 (AFBO + CTD)	5	120 mg/kg cimetidine + 200 mg/kg of aqueous extract	8 weeks treated
Group 5 (ELEBO + CTD)	5	120 mg/kg cimetidine + 200-mg/kg ethanol extract	8 weeks treated
Group 6 (BFBO + CTD)	5	120 mg/kg cimetidine + 200 mg/kg n-butanol fraction	8 weeks treated
Group 7 (HFBO + CTD)	5	120 mg/kg cimetidine + 200 mg/kg n-hexane fraction	8 weeks treated

Cimetidine was administered orally (daily) for 8 weeks. 1, Control; 2, ELEBO (ethanol leaf extract of *Brassica oleracea*); 3, CTD (cimetidine); 4, AFBO + CTD (aqueous fraction of *Brassica oleracea* + cimetidine); 5, ELEBO + CTD (ethanol leaf extract of *Brassica oleracea* + cimetidine); 6, BFBO + CTD (n-butanol fraction of *Brassica oleracea* + cimetidine); 7, HFBO + CTD (n-hexane fraction of *Brassica oleracea* + cimetidine).

### 2.9 Collard green acute toxicity

A young adult male Wistar rat was selected for the study and acclimatized under standard laboratory conditions, with unrestricted access to water and a standard pellet diet. Prior to the initiation of dosing, the animal underwent a fasting period of 3–4 h, during which water was made freely available. At the commencement of the acute oral toxicity evaluation, the rat, weighing 204 g, received a single oral dose of the test substance at the limit dose of 2,000 mg/kg body weight, administered via oral gavage using an appropriate cannula. The corresponding volume administered was 2 mL/kg body weight.

Post-administration, the animal was subjected to intensive monitoring for clinical signs of toxicity during the first 30 min, followed by periodic observations over the subsequent 24 h, and thereafter daily monitoring for a total of 14 days. Parameters assessed included signs of toxicity, behavioral changes, alterations in body weight, and mortality. Throughout the observation period, no clinical signs of toxicity or mortality were recorded.

Following the initial assessment and in accordance with the OECD guideline 425 (up-and-down procedure) (18), an additional four Wistar rats were recruited and subjected to the same dosing and observational regimen.

### 2.10 Weight calculation

The body weights of the rats were measured periodically throughout the study using a digital weighing scale. Following euthanasia, the testes, epididymis, and anterior pituitary gland were carefully excised, cleared of adherent adipose tissue, and weighed using a computerized analytical balance. The following was used to calculate relative organ weight (R_w_) and percentage weight change (%W) (19)

$$\%W=\frac{\left(\text{Final weight}-\text{Initial weight})\;(g\right)}{\text{Initial weight}\;(g)}\times 100$$

$$R_{w}=\frac{\text{Organ}\;\mathrm{wt}\;(g)}{\text{Body}\;\mathrm{wt}\;(g)}\times 100$$

### 2.11 Sacrifice method, organ collection, and disposal of waste

At the end of the 8-week treatment period, all experimental animals were anesthetized with a combination of ketamine (80 mg/kg) and xylazine (10 mg/kg) (20). The anesthetic agents were freshly prepared in sterile saline and administered intramuscularly using a 1 mL insulin syringe fitted with a 26-gauge needle. Blood samples were obtained via cardiac puncture and preserved for subsequent biochemical analyses. To obtain the serum, the blood samples were spun in a centrifuge for 5 min at 3,000 rpm. Following euthanasia, the testes, epididymis, and anterior pituitary glands were carefully dissected and weighed using a digital analytical balance for accurate mass determination. The right testis from each animal was immediately fixed in 10% neutral buffered formalin for histopathological processing and examination. All biological waste, including animal tissues and blood-contaminated materials, was disposed of through high-temperature incineration in compliance with established institutional biosafety and environmental safety protocols. This ensured the proper handling of potentially hazardous materials and minimized risks to personnel, public health, and the surrounding environment.

### 2.12 Hormonal assays

Using the enzyme-linked immunosorbent assay (ELISA) method and in accordance with the manufacturer’s instructions, the levels of luteinizing hormone, follicle-stimulating hormone, and testosterone in the serum was assessed (21).

#### 2.12.1 Luteinizing hormone assay

The rat luteinizing hormone (LH) Elisa kit (Catalogue CSB-E12654r Cusabio Wuhan, Hubei Province, China) was obtained and used for the analysis of LH according to manufacturer’s instruction. Briefly, all reagents were equilibrated to room temperature (approximately 22 °C) prior to use. The required number of wells was secured in a well holder. Subsequently, 50 μL each of standards, specimens, and controls were dispensed into the designated wells. To each well, 100 μL of enzyme conjugate reagent was added. The contents were thoroughly mixed using a microplate mixer for 30 s and incubated at 22 °C for 60 min in the dark. Following incubation, the plate contents were discarded by inverting the microplate over a sink. The wells were then washed five times using a microplate washer with the provided washing buffer. After the final wash, the plates were tapped onto absorbent tissue paper to remove residual liquid. Next, 100 μL of tetramethylbenzidine (TMB) substrate solution was added to each well, and the plate was gently mixed and incubated for 15 min. The enzymatic reaction was terminated by adding 50 μL of stop solution to each well, followed by gentle mixing for 30 s. A color change from blue to yellow indicated successful reaction termination. The optical density (OD) was measured using a microplate reader at a wavelength of 450/630 nm (21).

#### 2.12.2 Follicle stimulating hormone assay

The rat follicle-stimulating hormone, ELISA Kit (Catalogue CSB-E06869r Cusabio Wuhan, Hubei Province, China) was obtained and used for the analysis of FSH according to manufacturer’s instruction. Briefly, all reagents were brought to room temperature (approximately 22 °C) prior to use. The required number of wells was secured in a microplate well holder. Subsequently, 50 μL of standard, specimen, and control solutions were added to the designated wells. An enzyme conjugate reagent (100 μL) was then dispensed into each well. The contents of the wells were thoroughly mixed using a microplate mixer for 30 s, followed by incubation at 22 °C for 60 min. After incubation, the reaction mixture was discarded by inverting the plate over a sink. The microtiter wells were washed five times with the provided washing buffer using a microplate washer. After washing, the plate was gently tapped onto absorbent tissue paper to remove any residual liquid. Next, 100 μL of tetramethylbenzidine (TMB) substrate solution was added to each well, and the plate was gently mixed and incubated for 15 min. The reaction was terminated by adding 50 μL of stop solution to each well and mixing for 30 s. A color change from blue to yellow indicated the enzymatic reaction had occurred. The optical density (OD) was measured at 550/630 nm using a microplate reader (21).

#### 2.12.3 Testosterone assay

The Rat Free Testosterone, F-TESTO ELISA Kit hormone Elisa kit (Catalogue CSB-E05097r Cusabio Wuhan, Hubei Province, China) was obtained and used for the analysis of Testosterone according to manufacturer’s instruction. Briefly, a volume of 0.01 mL of serum was pipetted into the designated wells of the microplate. Subsequently, 0.05 mL of the working testosterone enzyme reagent was added to each well, and the plate was gently swirled for 20–30 s to ensure thorough mixing. Thereafter, 0.05 mL of the testosterone biotin reagent was added to all wells, followed by another gentle swirling for 20–30 s. The plate was then covered and incubated at room temperature (approximately 22 °C) for 60 min. Following incubation, the contents of the wells were discarded by decantation, and residual liquid was blotted using absorbent paper. The wells were washed three times with 0.35 mL of wash buffer per well, decanting and blotting the plate dry after each wash cycle. After washing, 0.1 mL of the working substrate solution was added to each well, and the plate was incubated at room temperature for 15 min. The enzymatic reaction was terminated by adding 0.05 mL of stop solution to each well, followed by gentle mixing for 15–20 s. All reagents were added in the same sequence across all wells to minimize variability in reaction timing. The optical density was measured at 450 nm using a microplate reader (Model SM600, Shanghai Yongchuang Medical Instrument Co., China) within 30 min of adding the stop solution. A standard dose–response curve was used to determine the concentration of testosterone in each sample (21).

### 2.13 Estimation of antioxidant (CAT and SOD) MDA levels

The estimation of the following antioxidants was assessed according to the following methods.

#### 2.13.1 Catalase

Catalase (CAT) activity was assessed using the method described by Sinha (22). Briefly, 0.5 mL of tissue supernatant was transferred into 15 mL ice-cold test tubes. The enzymatic reaction was initiated by the addition of 5 mL of 30 mM hydrogen peroxide (H₂O₂), followed by gentle mixing through tube inversion. After an incubation period of 3 min at room temperature, the reaction was terminated by adding 1 mL of 6 M sulfuric acid (H₂SO₄). Subsequently, 7 mL of 0.01 M potassium permanganate (KMnO₄) was added, and the absorbance was measured at 545 nm within 30–60 s using a spectrophotometer.

$$\text{Catalase activity}=\frac{\text{Absorbance}/\min\times V\times 1,000}{W\times M\times y}$$

V = total volume of the reaction mixture; W = weight of tissue; M = molar extinction coefficient which is 40.0; v = volume of sample used.

#### 2.13.2 Malondialdehyde

Malondialdehyde (MDA), a marker of lipid peroxidation, reacts with thiobarbituric acid (TBA) to form a pink chromogen with a maximum absorbance at 532 nm. The concentration of MDA in the tissue supernatant was determined using the method described by Prabhakar et al. (23). Briefly, 200 μL of clear testicular homogenate was mixed with 2 mL of a TBA-trichloroacetic acid (TCA) reagent, composed of 0.375% TBA and 15% TCA. The total volume was adjusted to 3 mL using double-distilled water. The mixture was incubated in a water bath at 95 °C for 20 min and then rapidly cooled under running tap water. Following cooling, 3 mL of n-butanol was added to extract the MDA-TBA adduct. The absorbance of the resulting pink-colored n-butanol extract was measured at 532 nm using a BSSUV-202 spectrophotometer. Each sample was analyzed in triplicate to ensure reproducibility, and MDA levels were quantified using a molar extinction coefficient of 1.56 × 10^5^ M^−1^ cm^−1^. Results were expressed as nanomoles (nmol) of MDA per gram of tissue.

$$\begin{array}{l} \text{Concentration of}\;\mathrm{MDA}\;(\text{nmole}/g\;\text{of tissue})\\ =\frac{\text{Absorbance of}\;\mathrm{TBA}-\mathrm{MDA}\;\text{complex}\;\mathrm{at}\;532\;\mathrm{nm}}{\text{Molar extinction coefficient of}\;\mathrm{MDA}\times \text{weight of tissue}} \end{array}$$

#### 2.13.3 Superoxide dismutase

Superoxide dismutase (SOD) catalyzes the dismutation of superoxide radicals into molecular oxygen and hydrogen peroxide. The enzymatic activity was determined based on SOD’s ability to inhibit the autoxidation of epinephrine (adrenaline) to adrenochrome, as described by Misra and Fridovich (24).

To initiate the assay, 2.0 mL of tissue supernatant was mixed with 2.5 mL of 0.05 M sodium carbonate buffer (pH 10.2) in a cuvette. The reaction was initiated by the rapid addition of 0.3 mL of freshly prepared 0.3 mM adrenaline solution, followed by thorough mixing. A reference (blank) cuvette contained 2.5 mL of the same carbonate buffer, 0.3 mL of adrenaline, and 0.2 mL of distilled water (in place of the sample). The formation of adrenochrome was monitored by measuring the increase in absorbance at 480 nm using a spectrophotometer. Absorbance readings were recorded at 30 s and again at 150 s. SOD activity was calculated based on the degree of inhibition of adrenaline autoxidation, using the appropriate equation as described in the original method.

$$\text{Increase in}\;\mathrm{Abs}\;\mathrm{per}\;\min=\frac{A_{1}-A_{0}}{2.5}$$

A_0_ = Abs after 30 s, A_1_ = Abs after 150 s.

$$\begin{array}{l} \%=\mathrm{age}\;\text{inhibition}\\ =\frac{\text{increase in}\;\mathrm{Abs}/\min\;\text{of reference}-\text{increase in}\;\mathrm{Abs}/\min\;\text{of adreno}\times 100}{\text{increase in}\;\mathrm{Abs}/\min\;\text{of reference}} \end{array}$$

where one unit of SOD activity was defined as the amount of SOD required to inhibit the auto-oxidation of adrenaline to adrenochrome by 50% for 1 min.

### 2.14 Histology of the testis, epididymis

The testes and epididymis were dissected out carefully and fixed in 10% neutral buffered formalin for 48 h. They were processed for histological examination following standard histological protocols (25, 26). Sections were cut with microtome (5 μm thick) and stained with H&E. The slides were then mounted for microscopic examination. Photomicrographs were viewed using the computer software.

### 2.15 Statistical analysis

One-way analysis of variance (ANOVA) was used for data analysis with the aid of GraphPad software version 8. Comparison was done using Tukey test. Results were expressed as mean ± S.E.M. *p* < 0.05 was taken as accepted level of significant difference.

## 3 Results

**Figure 2:**
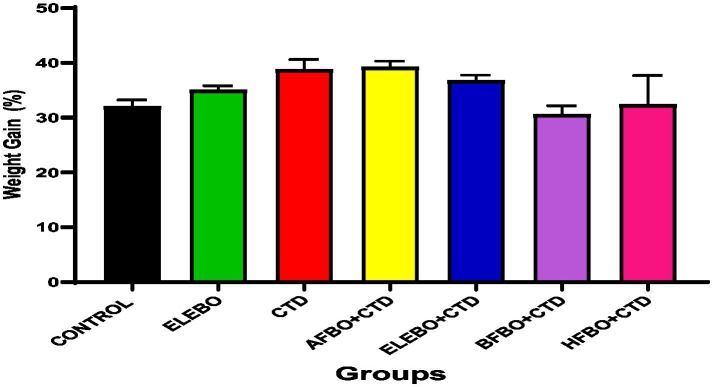
Effects of ethanol extract and solvent fractions of *Brassica oleracea* var. *viridis* (collard greens) on body weight gain in rats treated with cimetidine. No statistically significant differences in body weight gain were observed among the groups treated with cimetidine or the various extract fractions compared to the control group. Data are expressed as mean ± S.E.M (*n* = 5). Differences were considered statistically significant at *p* < 0.05. A-Control, ELEBO (ethanol leaf extract of *Brassica oleracea*), CTD (cimetidine), AFBO + CTD (aqueous fraction of *Brassica* oleracea + cimetidine) ELEBO + CTD (ethanol leaf extract of *Brassica oleracea* + cimetidine), BFBO + CTD (n-butanol fraction of *Brassica oleracea* + cimetidine), HFBO+CTD (n-hexane fraction of *Brassica oleracea* + cimetidine).

**Figure 3:**
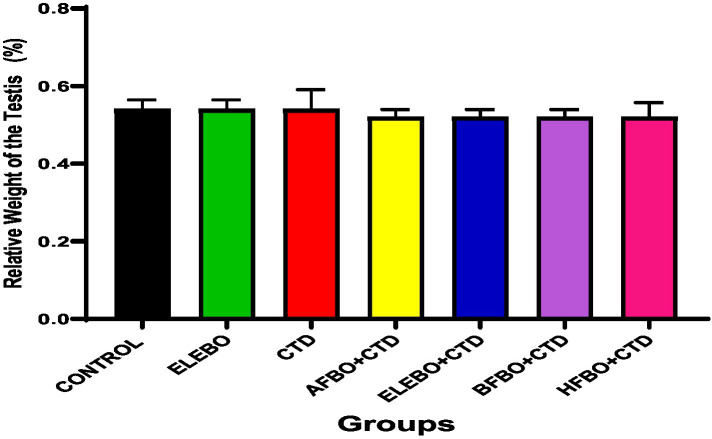
Effects of ethanol leaf extract and solvent fractions of *Brassica oleracea* var. *viridis* (collard greens) on relative weight of the testis of Wistar rats treated with cimetidine. There were no statistically significant differences in relative weight of the testis of Wistar rats observed among all groups following administration of cimetidine and the various extract fractions when compared with the control. Data are shown as mean ± S.E.M (*n* = 5). Differences were considered statistically significant at *p* < 0.05 A-Control, ELEBO (ethanol leaf extract of *Brassica oleracea*), CTD (cimetidine), AFBO + CTD (aqueous fraction of *Brassica oleracea* + cimetidine) ELEBO + CTD (ethanol leaf extract of *Brassica oleracea* + cimetidine), BFBO + CTD (n-butanol fraction of *Brassica oleracea* + cimetidine), HFBO + CTD (n-hexane fraction of *Brassica oleracea* + cimetidine).

**Figure 4:**
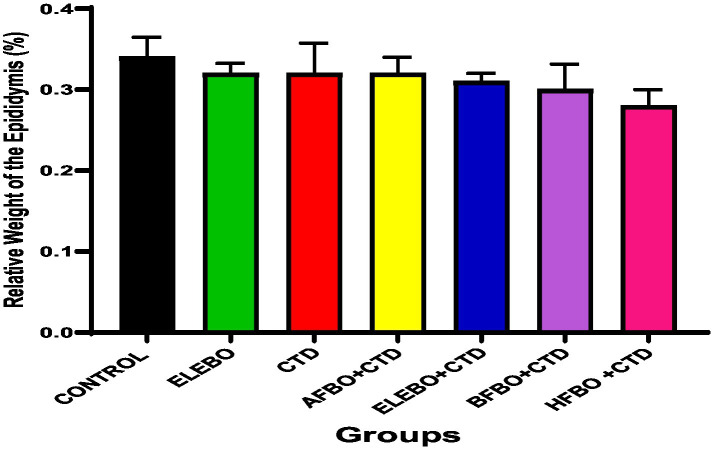
Effects of ethanol leaf extract and fractions of *Brassica oleracea* var. *viridis* (collard greens) on the relative weight of the epididymis of rats treated with cimetidine. There were no statistically significant differences in relative weight of the epididymis of Wistar rats observed among all groups following administration of cimetidine and the various extract fractions when compared with the control. Data are shown as mean ± S.E.M (*n* = 5). Mean values are considered to be significant at *p* < 0.05. A-Control, ELEBO (ethanol leaf extract of *Brassica oleracea*), CTD (cimetidine), AFBO + CTD (aqueous fraction of *Brassica oleracea* + cimetidine) ELEBO + CTD (ethanol leaf extract of *Brassica oleracea* + cimetidine), BFBO + CTD (n-butanol fraction of *Brassica oleracea* + cimetidine), HFBO + CTD (n-hexane fraction of *Brassica oleracea* + cimetidine).

**Figure 5:**
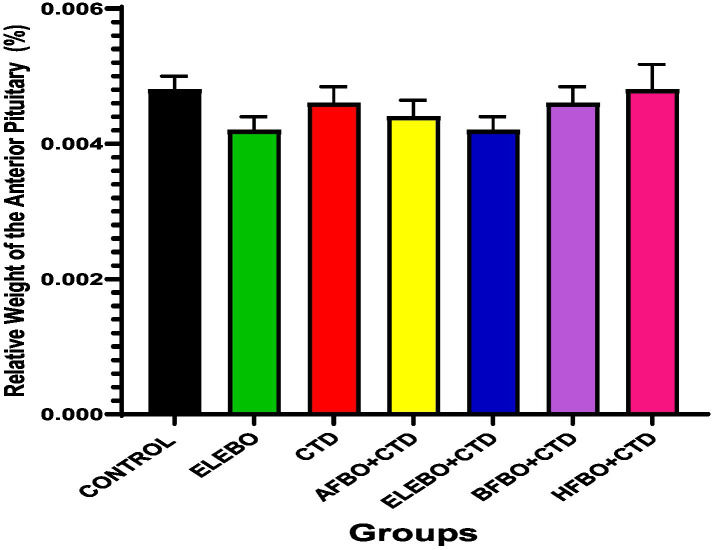
Effects of ethanol leaf extract and fractions of *Brassica oleracea* var. *viridis* (collard greens) on relative weight of anterior pituitary gland of rats treated with cimetidine. There were no statistically significant differences in relative weight of anterior pituitary gland of Wistar rats observed among all groups following administration of cimetidine and the various extract fractions when compared with the control. Data are shown as mean ± S.E.M (*n* = 5). Mean values are considered to be significant at *p* < 0.05. A-Control, ELEBO (ethanol leaf extract of *Brassica oleracea*), CTD (cimetidine), AFBO + CTD (Aqueous fraction of *Brassica oleracea* + cimetidine) ELEBO + CTD (ethanol leaf extract of *Brassica oleracea* + cimetidine), BFBO + CTD (n-butanol fraction of *Brassica oleracea* + cimetidine), HFBO + CTD (n-hexane fraction of *Brassica oleracea* + cimetidine).

**Figure 6:**
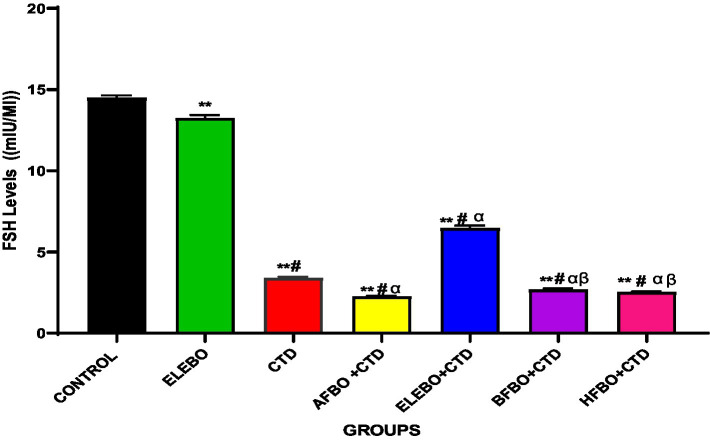
Effects of ethanol extract and fractions of *Brassica oleracea* var. *viridis* (collard greens) on serum level of FSH levels in rats treated with cimetidine. Data are shown as mean ± S.E.M (*n* = 5). Mean values were compared using Tukey’s multiple comparisons test, with significance set at *p* < 0.05. A significant decrease in FSH levels was observed in the cimetidine-treated group compared to the control. Similarly, FSH levels were significantly reduced in the AFBO + CTD, BFBO + CTD, and HFBO groups relative to the control. However, a slight increase in FSH levels was noted in the ELEBO + CTD group when compared with the other solvent fraction-treated groups. ** = *p* < 0.0001 compared with control, # = *p* < 0.0001 compared with ELEBO, *α* = *p* < 0.0001 compared with CTD, *β* = *p* < 0.0001 compared with ELEBO + CTD. A-Control, ELEBO (ethanol leaf extract of *Brassica oleracea*), CTD (cimetidine), AFBO + CTD (aqueous fraction of *Brassica oleracea* + cimetidine) ELEBO + CTD (ethanol leaf extract of *Brassica oleracea* + cimetidine), BFBO + CTD (n-butanol fraction of *Brassica oleracea* + cimetidine), HFBO + CTD (n-hexane fraction of *Brassica oleracea* + cimetidine).

**Figure 7:**
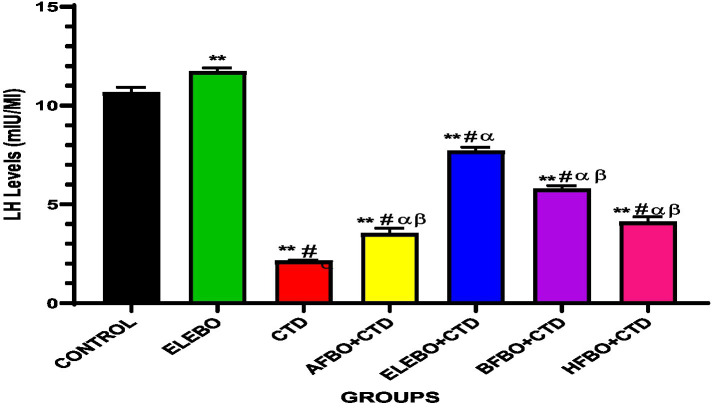
Effects of ethanol extract and fractions of *Brassica oleracea* var. *viridis* (collard greens) on serum level of LH levels in rats treated with cimetidine. Data are presented as mean ± S.E.M (*n* = 5). Mean values were compared using Tukey’s multiple comparisons test, with statistical significance set at *p* < 0.05. A significant decrease in LH levels was observed in the cimetidine-treated group compared to the control. However, this effect was reversed in the groups that received the various solvent fractions. Notably, the ELEBO-treated group showed a significant increase in LH levels compared to the other fraction-treated groups (*p* < 0.05). ** = *p* < 0.0001 compared with control, # = *p* < 0.0001 compared with ELEBO, *α* = *p* < 0.0001 compared with BFBO + CTD and ELEBO + CTD, *β* = *p* < 0.0001 compared with ELEBO + CTD. A-Control, ELEBO (ethanol leaf extract of *Brassica oleracea*), CTD (cimetidine), AFBO + CTD (aqueous fraction of *Brassica oleracea* + cimetidine) ELEBO + CTD (ethanol leaf extract of *Brassica oleracea* + cimetidine), BFBO + CTD (n-butanol fraction of *Brassica oleracea* + cimetidine), HFBO + CTD (n-hexane fraction of *Brassica oleracea* + cimetidine).

**Figure 8:**
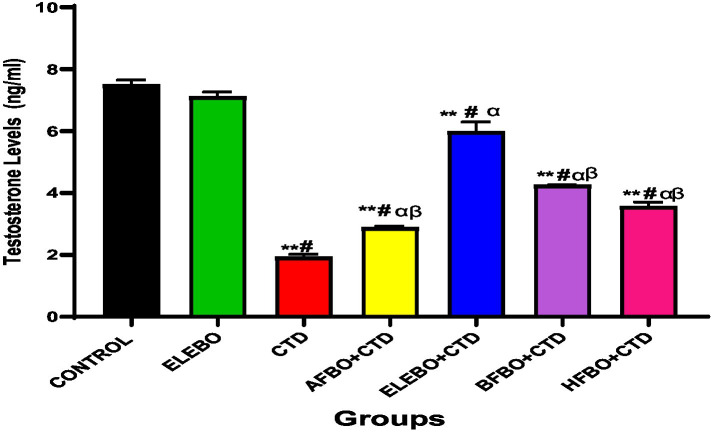
Effects of ethanol extract and solvent fractions of *Brassica oleracea* var. *viridis* (collard greens) on serum level of testosterone levels in rats treated with cimetidine. Data are presented as mean ± S.E.M (*n* = 5). Mean values were compared using Tukey’s multiple comparisons test, with statistical significance set at *p* < 0.05. A significant decrease in testosterone levels was observed in the cimetidine-treated group compared to the control. However, this effect was reversed in the groups that received the various solvent fractions. Among these, the ELEBO-treated group showed a significantly higher testosterone level compared to the other fraction-treated groups (*p* < 0.05). ** = *p* < 0.0001 compared with control, # = *p* < 0.0001 compared with ELEBO, *α* = *p* < 0.0001 compared with AFBO + CTD, HFBO + CTD and ELEBO + CTD group, *β* = *p* < 0.0001 compared with ELEBO + CTD. A-Control, ELEBO (ethanol leaf extract of *Brassica oleracea*), CTD (cimetidine), AFBO + CTD (Aqueous fraction of *Brassica oleracea* + cimetidine) ELEBO + CTD (ethanol leaf extract of *Brassica oleracea* + cimetidine), BFBO + CTD (n-butanol fraction of *Brassica oleracea* + cimetidine), HFBO + CTD (n-hexane fraction of *Brassica oleracea* + cimetidine).

**Figure 9:**
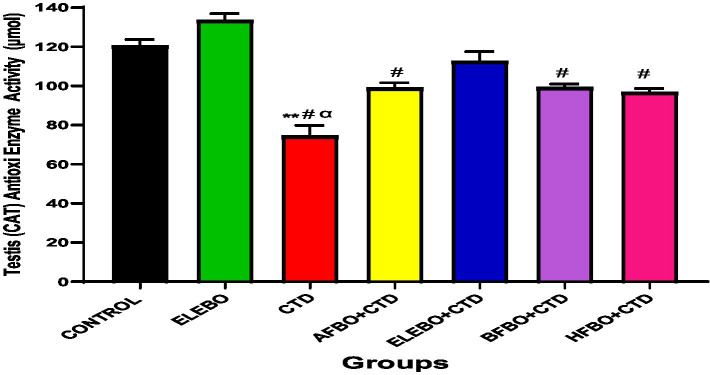
Effect of ethanol extract and solvent fractions of *Brassica oleracea* var. *viridis* (collard greens) on catalase activities in rats treated with cimetidine. Data are shown as mean ± S.E.M (*n* = 5). Mean values were compared among one another using the Turkey multiple comparisons test, revealing significant differences at *p* < 0.05, There was a significant decrease in CAT activities in the cimetidine treated group when compared with the control, however this effect was reversed in the groups that received ethanol extract and solvent fractions. ** = *p* < 0.0001 compared with control, # = *p* < 0.0001 compared with ELEBO, *α* = *p* < 0.0001 compared with ELEBO + CTD group. A-Control, ELEBO (ethanol leaf extract of *Brassica oleracea*), CTD (cimetidine), AFBO + CTD (aqueous fraction of *Brassica oleracea* + cimetidine) ELEBO + CTD (ethanol leaf extract of *Brassica oleracea* + cimetidine), BFBO + CTD (n-butanol fraction of *Brassica oleracea* + cimetidine), HFBO + CTD (n-hexane fraction of *Brassica oleracea* + cimetidine).

**Figure 10:**
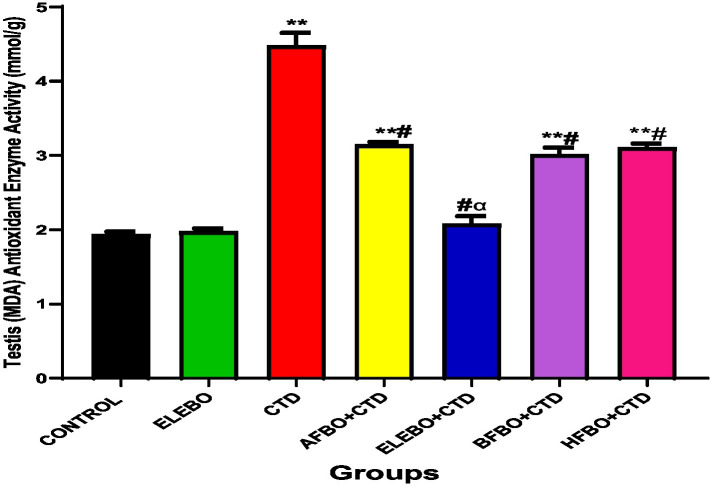
Effect of ethanol extract and fractions of *Brassica oleracea* var. *viridis* (collard greens) on MDA activities in rats treated with cimetidine. Data are shown as mean ± S.E.M (*n* = 5). “Mean values were compared using Tukey’s multiple comparisons test, with statistical significance set at *p* < 0.05. A significant increase in malondialdehyde (MDA) levels was observed in the cimetidine-treated group compared to the control. However, a significant decrease in MDA levels was observed in the ELEBO-treated group and in the groups that received the various solvent fractions when compared to the cimetidine group.” ** = *p* < 0.0001 compared with control, # = *p* < 0.0001 compared with CTD, *α* = *p* < 0.0001 compared with BFBO + CTD, AFBO + CTD and HFBO + CTD group. A-Control, ELEBO (ethanol leaf extract of *Brassica oleracea*), CTD (cimetidine), AFBO + CTD (aqueous fraction of *Brassica oleracea* + cimetidine) ELEBO + CTD (ethanol leaf extract of *Brassica oleracea* + cimetidine), BFBO + CTD (n-butanol fraction of *Brassica oleracea* + cimetidine), HFBO + CTD (n-hexane fraction of *Brassica oleracea* + cimetidine).

**Figure 11:**
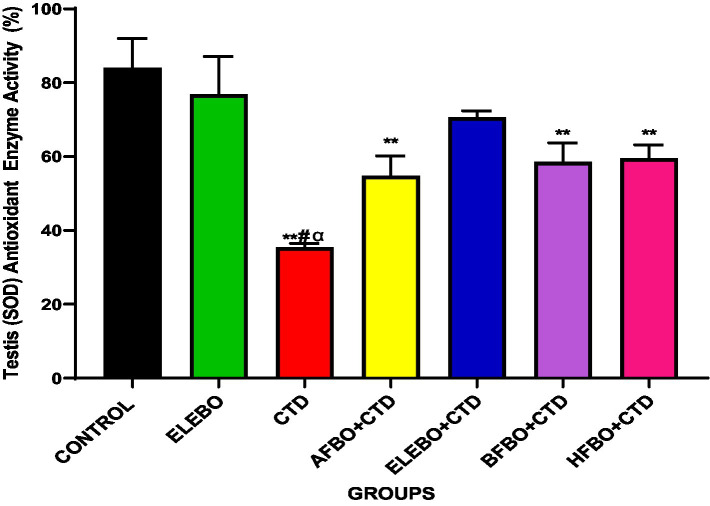
Effect of ethanol extract and fractions of *Brassica oleracea* var. *viridis* (collard greens) on superoxide dismutase activities in rats treated with cimetidine. Data are presented as mean ± S.E.M (*n* = 5). Mean values were compared using Tukey’s multiple comparisons test, with statistical significance set at *p* < 0.05. A significant decrease in superoxide dismutase (SOD) levels was observed in rats treated with cimetidine compared to the control group. However, a significant increase in SOD levels was observed in the ELEBO-treated group and in the groups that received the various solvent fractions (*p* < 0.05). ** = *p* < 0.0001 compared with control, # = *p* < 0.0001 compared with ELEBO, *α* = *p* < 0.0001 compared with BFBO + CTD, HFBO + CTD, AFBO + CTD and ELEBO group. A-Control, ELEBO (ethanol leaf extract of *Brassica oleracea*), CTD (cimetidine), AFBO + CTD (aqueous fraction of *Brassica oleracea* + cimetidine) ELEBO + CTD (ethanol leaf extract of *Brassica oleracea* + cimetidine), BFBO + CTD (n-butanol fraction of *Brassica oleracea* + cimetidine), HFBO + CTD (n-hexane fraction of *Brassica oleracea* + cimetidine).

**Figure 12:**
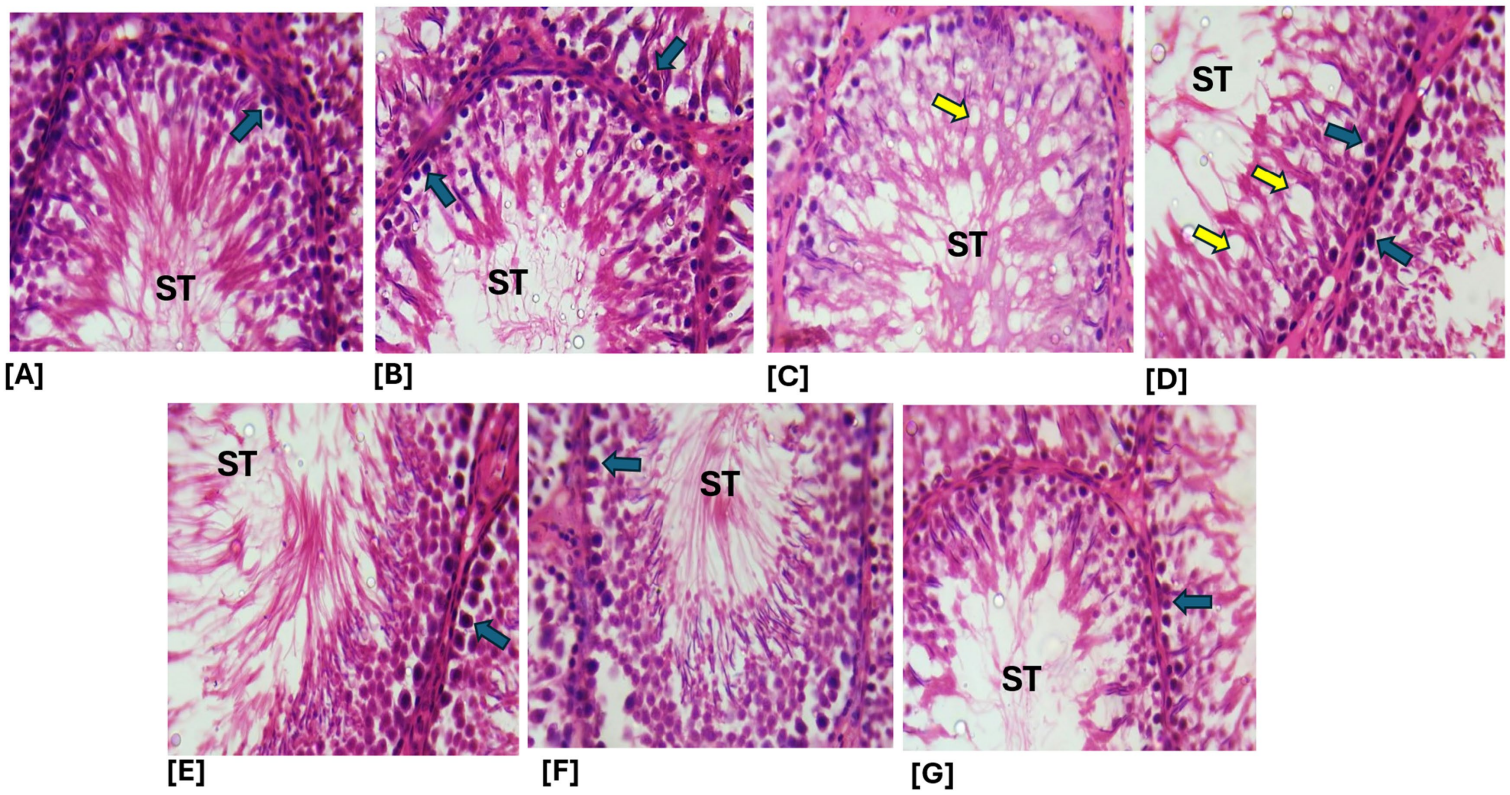
**(A,B)** Sections from the normal control and crude ethanol extract-treated groups showed relatively normal testicular histoarchitecture, with intact seminiferous tubules (ST) (blue arrows). **(C)** In contrast, sections from the cimetidine-treated group exhibited notable histopathological alterations, including vacuolation of the seminiferous tubules and disruption of the germ cell layers (yellow arrow). **(D–G)** However, sections from the groups treated with cimetidine in combination with the aqueous fraction, ethanol leaf extract, n-butanol fraction, and n-hexane fraction of *Brassica oleracea* displayed marked improvement in testicular histoarchitecture compared to the cimetidine-only group. **A**, Control; **B**, ELEBO treated group; **C**, CTD treated group; **D**, AFBO + CTD treated group; **E**, ELEBO + CTD extract treated group; **F**, BFBO + CTD extract treated group; **G**, HFBO + CTD extract treated group (H&E; ×250). AFBO (aqueous fraction of *Brassica oleracea*), CTD (cimetidine), ELEBO (ethanol leaf extract of *Brassica oleracea*), BFBO (n-butanol fraction of *Brassica oleracea*), HFBO (n-hexane fraction of *Brassica oleracea*) (H&E; ×400).

**Figure 13:**
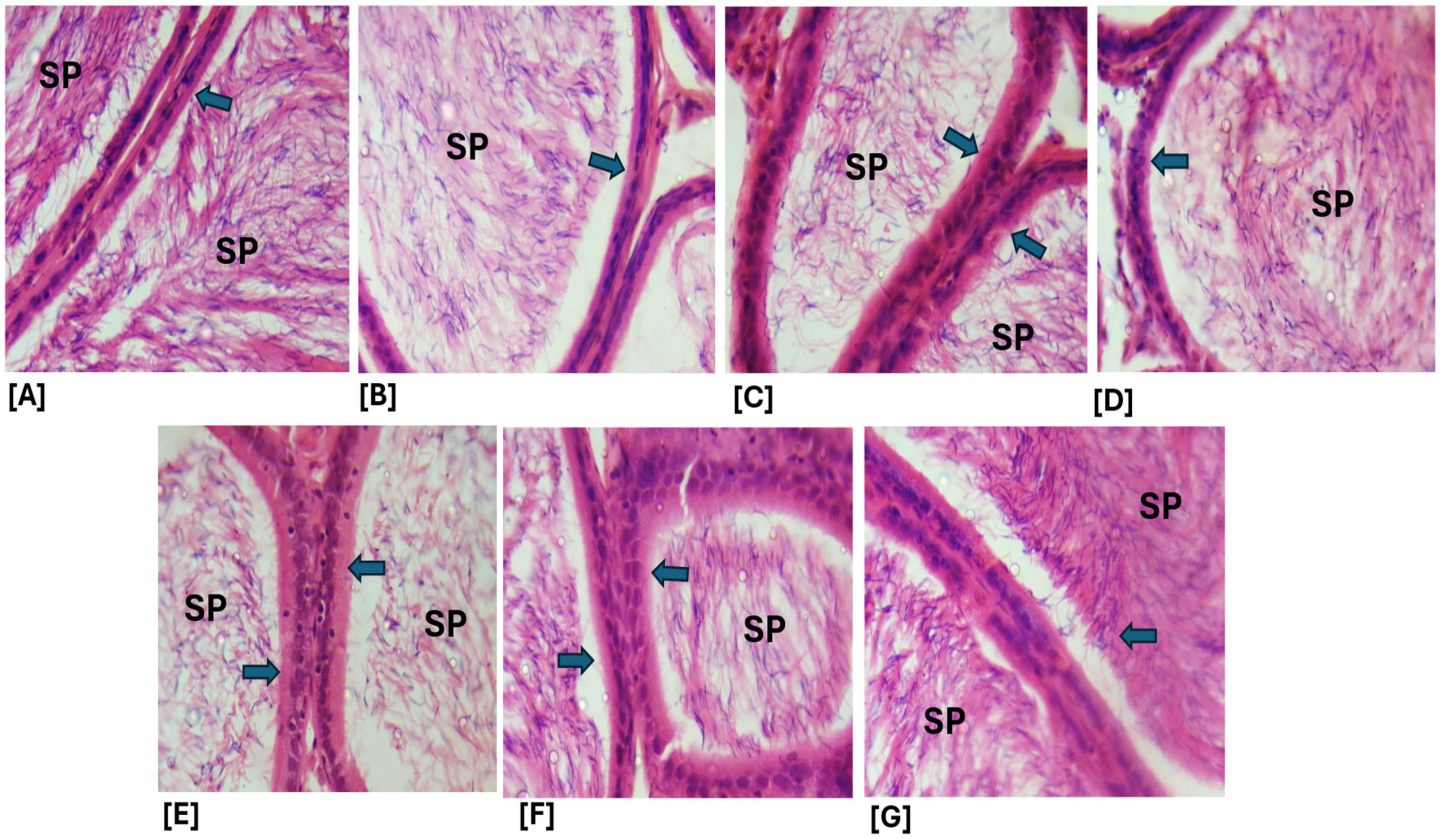
**(A–G)** Photomicrographs of epididymal sections from the various treatment groups show spermatozoa (SP) and the columnar epithelial lining of the epididymis (blue arrows). All treatment groups exhibited relatively intact histoarchitecture, with no observable lesions in the epithelial lining. **A**, Control; **B**, ELEBO treated group; **C**, CTD treated group; **D**, AFBO + CTD treated group; **E**, ELEBO + CTD extract treated group; **F**, BFBO + CTD extract treated group; **G**, HFBO + CTD extract treated group. AFBO (aqueous fraction of *Brassica oleracea*), CTD (cimetidine), ELEBO (ethanol leaf extract of *Brassica oleracea*), BFBO (n-butanol fraction of *Brassica oleracea*), HFBO (n-hexane fraction of *Brassica oleracea*) (H&E; ×400).

Effects of ethanol extract and fractions of *Brassica oleracea* var. *viridis* (Collard Greens) on relative weights of the testes, epididymis, and anterior pituitary gland, and body weights in male Wistar are shown in Figures 2–5. Determine the serum levels of steroid androgens (LH, FSH, testosterone) and antioxidant enzyme activities (catalase and superoxide dismutase) and malondialdehyde in male Wistar rats following treatment with ethanol extract and fractions of *Brassica oleracea* var. *viridis* as showed in Figures 6–11. Effect of ethanol extract and fractions of *Brassica oleracea* var. *viridis* (Collard Greens) on histology of the testis and epididymis in male Wistar rats with cimetidine-induced reproductive toxicity in male Wistar rats as shown in Figures 12, 13.

## 4 Discussion

This study evaluated the protective effects of the ethanol extract and solvent fractions of *Brassica oleracea* var. *viridis* (collard greens) against cimetidine-induced reproductive toxicity in male Wistar rats. Cimetidine, an H₂ receptor antagonist used for peptic ulcer treatment, is known to disrupt male reproductive function through antiandrogenic activity, oxidative stress, and histological damage to testicular tissue. The administration of *Brassica oleracea* var. *viridis* (collard greens) ethanol extract and solvent fractions was investigated as a potential therapeutic intervention due to its rich phytochemical profile, including antioxidants, flavonoids, and sterols (12).

This study evaluated the effects of *Brassica oleracea* var. *viridis* (collard greens) ethanol extract (ELEBO) and its solvent fractions on follicle-stimulating hormone (FSH) levels in male rats exposed to cimetidine, a well-known testicular toxicant that impairs spermatogenesis. FSH plays a vital role in regulating sperm production by stimulating Sertoli cells within the testes, and reductions in FSH are linked to compromised spermatogenesis and male infertility (6). Consistent with prior research, cimetidine administration significantly decreased FSH levels, confirming its gonadotoxic effects through disruption of hormonal balance and testicular function (27).

Importantly, co-treatment with ELEBO, both alone and combined with cimetidine (ELEBO + CTD), significantly attenuated the suppression of FSH induced by cimetidine. This restorative effect likely stems from the antioxidant-rich phytochemicals in *Brassica oleracea*, (collard greens) including flavonoids and glucosinolates, which may counteract the oxidative stress central to cimetidine’s testicular toxicity. These findings highlight the therapeutic potential of ELEBO in managing drug-induced male infertility. Notably, the aqueous, butanol, and hexane solvent fractions failed to replicate the full extract’s protective effects, suggesting that the bioactive compounds responsible for hormonal restoration either act synergistically or are more concentrated in the whole ethanol extract. Fractionation may have diluted these compounds or isolated less effective constituents, reducing efficacy.

These results emphasize the critical role of phytochemical synergy in *Brassica oleracea* var. *viridis* (collard greens) ethanol extract and solvent fractions protective action, aligning with findings by Abdel-Aty et al. (28), who reported that crude aqueous *Moringa oleifera* extract similarly restored FSH and testosterone levels in monosodium glutamate-induced testicular toxicity in adult male albino rats. This parallel supports the growing evidence that plant-based extracts rich in diverse bioactives can facilitate hormonal recovery following toxic insults.

The study also demonstrated that luteinizing hormone (LH) levels, which are essential for stimulating Leydig cells to produce testosterone and maintain spermatogenesis, were significantly reduced by cimetidine. ELEBO and ELEBO + CTD co-treatment effectively restored LH levels, suggesting a positive effect on pituitary-gonadal axis function and Leydig cell activity. Although partial LH restoration was observed with the solvent fractions (AFBO, BFBO, and HFBO), the whole ethanol extract produced the most pronounced effect, further supporting the hypothesis of synergistic interactions among bioactive compounds in the full extract.

Consistent with these hormonal improvements, testosterone levels suppressed by cimetidine were significantly restored in both the full extract and solvent fraction groups. Cimetidine’s inhibition of Leydig cell function and androgen biosynthesis is well documented (6), and the ability of *Brassica oleracea* var. *viridis* (collard greens) ethanol extract and solvent fractions to reverse this suppression underscores their potential as anti-infertility agents. Among the fractions, ELEBO + CTD and BFBO + CTD showed comparable efficacy, potentially reflecting the enhanced bioavailability and tissue penetration of lipophilic compounds in the butanol fraction.

These findings concur with previous studies on natural interventions for drug-induced reproductive toxicity. For example, Kolawole et al. (29) demonstrated that *Brassica oleracea* (cabbage) protected against lead-induced testicular damage and restored reproductive hormones. Collectively, the evidence suggests that *Brassica oleracea* var. *viridis* (collard greens) exerts its spermatoprotective effects through antioxidant activity and hormonal modulation, making it a promising candidate for managing male infertility associated with toxicant exposure.

Increased malondialdehyde (MDA) levels in the cimetidine-only group further confirm elevated lipid peroxidation, reinforcing the link between oxidative stress and cimetidine-induced testicular toxicity. Notably, co-treatment with *Brassica oleracea* var. *viridis* (collard greens) extracts and solvent fractions significantly reduced MDA concentrations, indicating mitigation of oxidative damage. These results align with previous studies demonstrating that *B. oleracea* (broccoli and caraway extract) not only reduce oxidative biomarkers but also improve sperm quality, testicular structure, and antioxidant enzyme activity (30). The present study demonstrates that cimetidine administration significantly suppresses catalase (CAT) activity in male Wistar rats, indicating an increase in oxidative stress within testicular tissue. Co-administration with ethanol extract and solvent fractions of *Brassica oleracea* var. *viridis* effectively reversed this suppression, suggesting that the plant extract exerts a protective antioxidant effect. Since catalase plays a vital role in detoxifying hydrogen peroxide and maintaining redox balance, its inhibition by cimetidine can result in the accumulation of reactive oxygen species (ROS), leading to lipid peroxidation, germ cell apoptosis, and impaired spermatogenesis.

Although some reports suggest that cimetidine-induced DNA damage may not always result in overt infertility in rats, oxidative stress remains a recognized contributor to male reproductive dysfunction (31). The protective effect observed in this study is consistent with previous findings highlighting the antioxidative potential of *B. oleracea* (broccoli methanol extract), which is rich in flavonoids, vitamins C and E, selenium, and other phytochemicals with established antioxidant properties (32).

This study reported a significant decline in superoxide dismutase (SOD) activity following reproductive toxicity with cimetidine which was reversed upon administration of *Brassica oleracea* var. *viridis* (collard greens) ethanol extract and solvent fractions. SOD is essential for dismutating superoxide radicals into less harmful species. The elevation of its level following administration of *Brassica oleracea* var. *viridis* (collard greens) extract and fractions further supports the antioxidant efficacy of the extract. The cumulative effect of restoring both CAT and SOD activities underlines the extract’s role in protecting testicular function and improving sperm parameters, including motility, viability, and morphology. Overall, these findings underscore the therapeutic potential of *Brassica oleracea* var. *viridis* ethanol extract and solvent fractions in ameliorating cimetidine-induced oxidative damage and preserving male reproductive health.

Microscopic examination of testicular tissue sections provides critical insights into the protective and restorative effects of *Brassica oleracea* var. *viridis* (collard greens) ethanol extract and solvent fractions on testicular tissue. The cimetidine-treated group (CTD) exhibited areas of Histopathological alterations, evident as vacuolation of seminiferous tubule and disruption of germ cell layers, indicating testicular toxicity characterized by germinal epithelium degeneration and decreased spermatogenic activity. These findings align with oral cimetidine induced pathological changes in the testicles and hormone secretion disorder in rats. COX-2, iNOS, and NF-κB upregulation and induction of apoptosis may be associated with the reproductive toxicity caused by cimetidine, as previously reported by Liu et al. (17).

The groups co-administered cimetidine with the ethanol extract (ELEBO), aqueous (AFBO), n-butanol (BFBO), and n-hexane (HFBO) fractions of *Brassica oleracea* var. *viridis* (collard greens) demonstrated marked improvement in testicular histoarchitecture. The seminiferous tubules appeared more intact and cellular compared to the cimetidine-only group, suggesting a protective or regenerative role of the plant-based treatments. This observation is consistent with reports from previous studies that *B. oleracea* (cabbage) protected the testis against drug-induced oxidative stress and toxicity (29). The phytochemicals found in *Brassica oleracea* var. *viridis* (collard greens) ethanol extract and solvent fractions could reduce oxidative stress through compounds such as quercetin and gamma-sitosterol, kaempferol, and other antioxidant flavonoids (12).

Protecting the integrity of seminiferous tubules is crucial in male reproduction. Damage to these structures often results in poor sperm quality and reduced fertility. The enhancement observed in the treated groups demonstrates that *Brassica oleracea* var. *viridis* (collard greens) ethanol extract and solvent fractions may function as a potential agent to preserve testicular integrity and potentially reverse drug-induced infertility. A previous finding reported that *B. oleracea* (cabbage) protects testicular tissue due to its antioxidant and anti-inflammatory compounds (29).

The epididymal tissue sections from all treatment groups, including cimetidine-treated animals, demonstrated normal structural organization with unaltered epithelial tissue integrity. The columnar epithelial structure and presence of spermatozoa in the lumen indicate that the extract and its fractions protected the epididymis’ functional capacity during cimetidine exposure. This observation is significant because the epididymis plays a vital role in sperm maturation, storage, and transport. Impairment of sperm maturation due to structural damage to the epididymis can reduce fertilizing capacity (33). The protective properties of *Brassica oleracea* var. *viridis* (collard greens) ethanol extract and solvent fractions are evident as all extract-treated groups showed minimal testicular damage. The sperm maturation process becomes defective when testicular structural alterations reduce fertilization ability. The post-testicular activity of cimetidine may have been mitigated by *Brassica oleracea* var. *viridis* (collard greens) ethanol extract and solvent fractions, as all extract-treated groups showed minimal histopathological changes. This suggests that *B. oleracea* phytocompounds may safeguard epididymal function by minimizing oxidative tissue damage (34). The epididymis demonstrated less sensitivity to cimetidine damage than testicular tissue in this study probably due to tissue-specific protective mechanisms. The present *in vivo* study demonstrated that the administration of *Brassica oleracea* var. *viridis* (collard greens) ethanol extract and solvent fractions (ethanol, aqueous, n-butanol, and n-hexane), either alone or in combination with cimetidine (CTD) for 8 weeks, did not result in statistically significant alterations in body weight gain among the treated Wistar rats. This finding suggests that the reproductive toxicity associated with cimetidine operates independently of systemic changes in body weight.

Cimetidine, a histamine H₂-receptor antagonist, is well-documented to exert reproductive toxicity in males, primarily through its antiandrogenic effects, without significantly affecting body mass. This aligns with findings from Liu et al. (17), who reported that oral administration of cimetidine at doses of 20, 40, or 120 mg/kg/day for 9 weeks significantly impaired sperm motility and increased testicular apoptosis without notable changes in body weight. These findings reinforce the concept that cimetidine’s adverse reproductive effects are not necessarily linked to alterations in body mass, highlighting the importance of assessing specific reproductive endpoints beyond general growth metrics.

The absence of significant changes in body weight among rats treated with *B. oleracea* extracts, alone or in combination with cimetidine, suggests that these extracts neither potentiated nor mitigated cimetidine’s systemic metabolic effects. However, *Brassica oleracea* is known to contain phytochemicals such as glucosinolates, flavonoids, and sterols, which have been associated with protective effects against reproductive toxicity (12). This study is consistent with that of Patil et al. (35), who reported that hydroalcoholic extracts of *Brassica oleracea* L. var. *italica* (broccoli) enhanced fertility in both normal and HCG-induced ovulation models in rodents. While our data suggest that these extracts do not influence body weight, they may offer functional protection against cimetidine-induced reproductive toxicity at the cellular or endocrine level.

Further analysis of the relative weight of the epididymis revealed no significant differences among the experimental groups (*p* > 0.7147), including those receiving cimetidine alone or in combination with *Brassica oleracea* var. *viridis* (collard greens) ethanol extract and solvent fractions. The lack of observable structural changes in the epididymis suggests that neither cimetidine nor the plant extracts caused gross anatomical damage. However, organ weight alone is not a definitive indicator of reproductive function. Previous studies such as Liu et al. (17), have shown that cimetidine reduces sperm count and motility without affecting organ mass. These findings are consistent with those of Kolawole et al. (29), who reported that *Brassica oleracea* (cabbage) effectively mitigated lead acetate-induced testicular toxicity by reducing oxidative stress, restoring hormonal balance, and enhancing sperm quality. Their study reinforces the therapeutic potential of *Brassica oleracea* var. *viridis* (collard greens) ethanol extract and solvent fractions in supporting male reproductive health through its antioxidant and endocrine-modulating properties. While this provides a strong foundation for its broader reproductive benefits, additional research is necessary to specifically elucidate its protective mechanisms against cimetidine-induced reproductive dysfunction.

Similarly, no significant differences were observed in the relative weights of the testes across all groups, indicating that cimetidine and *Brassica oleracea* var. *viridis* (collard greens) ethanol extract and solvent fractions did not induce testicular atrophy or hypertrophy. This finding complements the epididymis data and further supports the notion that gross anatomical damage was not evident. These results corroborate those of Adelakun et al. (36), who noted that although cimetidine impairs Leydig cell function and disrupts testosterone synthesis, such effects may occur without changes in testicular weight.

The relative weight of the anterior pituitary gland remained unchanged across all treatment groups. As the anterior pituitary regulates male reproductive function by secreting luteinizing hormone (LH) and follicle-stimulating hormone (FSH), which, respectively, stimulate testosterone production and support spermatogenesis, functional disruptions at this level may impair fertility even in the absence of morphological alterations. The absence of pituitary weight changes suggests that *Brassica oleracea* var. *viridis* (collard greens) ethanol extract and solvent fractions did not modulate cimetidine’s effects on pituitary mass.

This interpretation is supported by findings from Adelakun et al. (36) who demonstrated that cimetidine indirectly impairs pituitary function by suppressing hypothalamic signaling, leading to altered gonadotropin (LH/FSH) release without changes in gland morphology. The antioxidant and phytoestrogenic constituents of *Brassica oleracea* var. *viridis* (collard greens) ethanol extract and solvent fractions may contribute to endocrine homeostasis and provide a degree of functional stability.

## 5 Conclusion

This study demonstrates that *Brassica oleracea* var. *viridis* ethanol extract and its solvent fractions possess significant protective effects against cimetidine-induced reproductive toxicity in male rats. These effects are mediated via Restoration of FSH, LH, and testosterone levels, Reduction of oxidative stress through enhancement of CAT and SOD activities, amelioration of histopathological damage in testicular tissue, preservation of epididymal architecture and function. The ethanol extract exhibited the most robust protective effect, underscoring the importance of phytochemical synergy. These findings suggest that *Brassica oleracea* var. *viridis* (collard greens) ethanol extract and solvent fractions may serve as a promising nutraceutical or adjunct therapy for managing drug-induced male infertility. However, further studies are warranted to isolate active compounds, elucidate molecular mechanisms, and assess long-term safety and efficacy in clinical settings.

### 5.1 Recommendation

Given the promising findings of this study, it is recommended that future research focus on further elucidating the mechanisms underlying the protective effects of *Brassica oleracea* var. *viridis* ethanol extract on male reproductive health. Specifically, detailed phytochemical analyses should be conducted to identify and isolate the active constituents responsible for the observed antioxidant and endocrine-modulating activities. Understanding the precise molecular targets and signaling pathways involved could enhance the therapeutic application of this plant.

In addition, long-term toxicity studies and pharmacokinetic profiling are essential to evaluate the safety, optimal dosage, and bioavailability of the extract when used chronically. This will be crucial in advancing the extract from experimental use to clinical application. Furthermore, controlled clinical trials involving human participants are necessary to validate the efficacy of *B. oleracea* in preventing or reversing drug-induced reproductive toxicity, particularly in individuals undergoing treatments known to impair fertility, such as cimetidine or other antiandrogenic medications.

Finally, the development of standardized nutraceutical formulations containing *Brassica oleracea* var. *viridis* (collard greens) ethanol extract and solvent fractions could offer a practical and accessible preventive strategy for maintaining male reproductive health. These efforts would contribute to the growing field of plant-based therapeutics and support the integration of evidence-based herbal medicine into conventional healthcare practices.

### 5.2 Limitations of the study

While this study provides valuable insights into the biological effects and phytochemical potential of *Brassica oleracea* extracts, certain limitations should be acknowledged. First, the study was conducted using an animal model, which may not fully replicate human physiological responses; therefore, extrapolation to clinical settings should be done cautiously. Second, the study focused primarily on ethanol-based extraction and a limited range of solvent fractions, potentially overlooking other bioactive compounds present in different polarities or extraction methods. Third, due to resource constraints, advanced molecular analyses (e.g., gene expression profiling or pathway-specific assays) were not included, which could have provided deeper mechanistic insights. Lastly, the study was conducted under controlled laboratory conditions, and environmental or dietary factors that may influence outcomes in real-world settings were not considered.

Future research should include broader extraction techniques, extended biochemical profiling, and translational studies to validate these findings in human subjects.

## Data Availability

The original contributions presented in the study are included in the article/supplementary material, further inquiries can be directed to the corresponding author.
